# Excess Folic Acid Supplementation before and during Pregnancy and Lactation Alters Behaviors and Brain Gene Expression in Female Mouse Offspring

**DOI:** 10.3390/nu14010066

**Published:** 2021-12-24

**Authors:** Xingyue Yang, Wenyan Sun, Qian Wu, Hongyan Lin, Zhixing Lu, Xin Shen, Yongqi Chen, Yan Zhou, Li Huang, Feng Wu, Fei Liu, Dandan Chu

**Affiliations:** 1 Key Laboratory of Neuroregeneration of Jiangsu and Ministry of Education, NMPA Key Laboratory for Research and Evaluation of Tissue Engineering Technology Products, Co-Innovation Center of Neuroregeneration, Nantong University, Nantong 226001, China; yangxingyue618@163.com (X.Y.); wq15706295797@163.com (Q.W.); sx827477223@outlook.com (X.S.); Huangli@ntu.edu.cn (L.H.); 2 Department of Pharmacology, School of Pharmacy, Nantong University, Nantong 226001, China; LHY031583@163.com (H.L.); wf619@ntu.edu.cn (F.W.); 3 Department of Biochemistry and Molecular Biology, School of Medicine, Nantong University, Nantong 226001, China; sunjiajun51601@163.com (W.S.); Tsukiato0412@gmail.com (Z.L.); cyq1530852896@163.com (Y.C.); ntzhouy@ntu.edu.cn (Y.Z.); 4 Department of Neurochemistry, Inge Grundke-Iqbal Research Floor, New York State Institute for Basic Research in Developmental Disabilities, Staten Island, NY 10314, USA

**Keywords:** folic acid, pregnancy, lactation, behaviors, gene expression, female

## Abstract

Use of folic acid (FA) during early pregnancy protects against birth defects. However, excess FA has shown gender-specific neurodevelopmental toxicity. Previously, we fed the mice with 2.5 times the recommended amount of FA one week prior to mating and during the pregnancy and lactation periods, and detected the activated expression of *Fos* and related genes in the brains of weaning male offspring, as well as behavioral abnormalities in the adults. Here, we studied whether female offspring were affected by the same dosage of FA. An open field test, three-chamber social approach and social novelty test, an elevated plus-maze, rotarod test and the Morris water maze task were used to evaluate their behaviors. RNA sequencing was performed to identify differentially expressed genes in the brains. Quantitative real time-PCR (qRT-PCR) and Western blots were applied to verify the changes in gene expression. We found increased anxiety and impaired exploratory behavior, motor coordination and spatial memory in FA-exposed females. The brain transcriptome revealed 36 up-regulated and 79 down-regulated genes in their brains at weaning. The increase of *Tlr1*; *Sult1a1*; *Tph2*; *Acacb*; *Etnppl*; *Angptl4* and *Apold1*, as well as a decrease of *Ppara* mRNA were confirmed by qRT-PCR. Among these genes; the mRNA levels of *Etnppl; Angptl4* *and* *Apold1* were increased in the both FA-exposed female and male brains. The elevation of Sult1a1 protein was confirmed by Western blots. Our data suggest that excess FA alteres brain gene expression and behaviors in female offspring, of which certain genes show apparent gender specificity.

## 1. Introduction

The water-soluble micronutrient folate (vitamin B-9) participates in multiple biological processes in mammals, such as DNA and protein synthesis, as well as nucleic acid and protein methylation [1]. As the fully oxidized synthetic form of natural folate, folic acid (FA) is catalyzed into 5-methyltetrahydrofolate (5-MTHF) in the liver, and finally produces the universal methyl donor S-adenosyl methionine (SAM), utilized in methylation reactions [1]. Appropriate FA supplement (0.4 mg per day) is widely accepted to prevent against neural tube defects (NTD) and other birth defects [2]. Moreover, FA fortification has been introduced in the United States, Canada and several other countries to diminish the number of NTD [3]. Due to vitamin supplements and mandatory fortification, high concentrations of folate have been detected in maternal serum as well as in breast milk [4,5,6,7]. Whether excess FA supplementation during pregnancy and lactation affects the neurodevelopment of offspring has become an important public health issue.

The negative impact of excessive maternal FA intake on infant neurodevelopment has been reported by several groups. Periconceptional use of FA exceeding the maximum tolerated dose (≥1 mg/day) was associated with decreased cognitive development in children [8]. FA supplementation during gestation is considered to be related to an increased risk for autism [9]. Extremely high concentrations of maternal plasma FA (>60.3 nmol/L) elevated the risk of autism spectrum disorder by 2.5 times [10,11]. In rodents, female mice supplemented with high dose of FA (40 mg/kg) before and during pregnancy leaded to embryonic delay and neural tube defects of the offspring [12]. Maternal over-supplementation with FA resulted in behavioral abnormalities in the offspring, such as anxiety-like behavior, hyperactivity, increased ultrasonic vocalizations [13] and seizure susceptibility [14], as well as impaired reversal learning [15]. The gene expression and DNA methylation profiling were altered in the brains of FA-exposed offspring [13,16,17,18]. Sex-specific influences of excess FA on behavioral defects of the offspring have been reported. Pregnant mice fed with 4 mg/kg diet of FA induced hyperactivity of male but not female offspring [13]. Maternal over-supplementation with FA was associated with attentional dysfunction in children, particularly in boys [19]. However, the roles of excess FA intake during the perigestational and lactational period on the brain transcriptome of weaning female mice and their behaviors after adulthood are not fully understood.

Previously, we fed the mice with approximately 2.5 times the recommended amount of FA one week prior to mating and during pregnancy and the lactation period, and detected behavioral defects in adult male offspring, including increased anxiety, impairments in social preference, and motor and spatial learning [18]. Transcriptome sequencing of the brains revealed that *Fos* and associated genes were upregulated in weaning male offspring. Here we applied the same dosage of FA to the mice and examined whether excess FA altered behaviors and gene expression in the brains of female offspring, and whether the changes are different from those in males. Behavior test showed that early exposure to excessive FA resulted in long-term alterations of behaviors in adult female offspring, such as increased anxiety-like behavior, decreased exploratory activities, motor coordination and spatial memory. RNA sequencing was used to identify differentially expressed genes (DEGs) in the brains of FA-exposed female mice at weaning. Quantitative real time-PCR (qRT-PCR) revealed the increased transcription of *Tlr1*, *Sult1a1*, *Tph2*, *Acacb*, *Etnppl*, *Angptl4* and *Apold1*, and decreased transcription of *Ppara* in FA-exposed female brains. Western blots confirmed the promoted expression of Sult1a1 protein FA-exposed brains. These results indicated that excess FA supplementation in pregnancy and lactation altered brain transcriptome in weaning female mice and changes adult behaviors, and that the effects were different from those of males.

## 2. Materials and Methods

### 2.1. FA Supplementation and Animals

The animal experiments were performed according to the National Institutes of Health Guidelines for animal research. ICR mice were purchased from Shanghai, China and maintained in the laboratory animal center of Nantong University. Mice were housed at 3–5 per cage on a 12 h light/12 h dark cycle with free access to food and water. The mice were supplied normal chow diet, containing the routinely recommended supplement of 2 mg FA/kg diet (Xietong Pharmaceutical Biotechnology, Nanjing, China) [20]. Formula of the diet is supplied in Supplementary Table S1. At 8 weeks of age, 4 males and 8 females were randomly divided into two groups and supplied with no (control) or 3.75 mg/L FA (Sigma-Aldrich, St. Louis, MO, USA) in their drinking water for one week [18,21,22]. Then, the mice were allowed to mate naturally by introducing 2 females in the cage of 1 male. FA was administrated throughout the mating, pregnancy and lactation period. A breeding mouse consumes about 5 g in their diet per day [23], which provides 10 µg FA. An adult mouse consumes an average of 4–8 mL of water daily [24], that is, at least 15 µg of FA from the drinking water. Therefore, the total amount of FA taken by the mice from food and water is at least 2.5 times (2.5 × FA) the normal dietary intake [18]. This dosage of FA was used because the total intake of folate from FA fortification and vitamin supplement exceeded twice the recommended dietary allowance (RDA) in the North American populations [25] and the serum levels of total folate increased approximately 2.5-fold after fortification in the U.S. population [6].

After weaning (postnatal day 21, P21d), the pups were supplied with standard chow and water. For RNA sequencing, qRT-PCR and Western blots, the pups were sacrificed at weaning. The cerebrums were quickly dissected, incubated in phosphate-buffered saline (PBS) on ice for 5 min [26], quickly frozen in liquid nitrogen and then stored at −80 °C before use. The body weight was recorded at 21 days and 1, 2, 3, 4 and 5 months of age (Con, *n* = 12; 2.5 × FA, *n* = 9).

### 2.2. Behavioral Analysis

All behavioral experiments were performed between 8 am and 5 pm. Female offspring were collected from 3–4 litters per group (2–5 females/litter). Two-month-old offspring were subjected to the behavioral tests in the following order: open field, three-chamber social approach and social novelty test, elevated plus-maze, rotarod test and Morris water maze task (Con, *n* = 12; 2.5 × FA, *n* = 9). Except for the rotarod, the behaviors were recorded and analyzed by a video-tracking system (ANYmaze, Stoelting Co., Wood Dale, IL, USA). The apparatuses were wiped with water first, then with 75% ethanol, and were allowed to dry between mice.

#### 2.2.1. Open Field Test

The mice were placed in a 48 cm L × 48 cm W × 40 cm H black plastic arena (Xinruan, Shanghai, China) and tracked every 2.5 min for 4 consecutive sessions, with a total time of 10 min. A 15 × 15 cm area in the center of the open field was designated as the central zone. The total distance, immobile time, mean speed, time in the central zone, entries into the central zone, and mean distance from the central zone of the mice were recorded.

#### 2.2.2. Three-Chamber Social Approach and Social Novelty Test

Sociability and social novelty of the mice were evaluated in a three-chambered box [27] (Ugo Basile, Gemonio, Italy), with each chamber being 20 cm W × 40 cm L × 22 cm H. The three chambers were separated by transparent Plexiglas walls with an 8 cm H × 5 cm W door in the middle. As described [18], the subject mouse was first introduced into the central chamber (Central) and allowed to habituate to the apparatus freely for 5 min. Then, a 2 month-old stranger female (Social) was settled in a wire cage and put in the left chamber. In the right chamber, an identical empty wire cage was placed as the inanimate target (Inanimate). The subject was allowed to explore the whole apparatus for 10 min. Time spent in each chamber was recorded to evaluate the sociability of the mice. In the sociability phase, another 2 month-old stranger female was enclosed in the cage in the right chamber (Novel). The subject was then allowed to explore the whole apparatus for another 10 min. The time spent exploring each chamber was calculated to evaluate the preference for social novelty.

#### 2.2.3. Elevated Plus-Maze

The elevated plus-maze apparatus was described previously [18]. The subject was introduced in the central square facing a closed arm, and allowed to explore the elevated plus-maze for 5 min. The time of mice exploring the closed or open arms were recorded to access anxiety-like behavior.

#### 2.2.4. Rotarod

The female offspring were accessed on the rotarod (Ugo Basile, Gemonio, Italy) 3 trails per day for 5 consecutive days. Rotarod steadily accelerated from 4 revolutions per minute (rpm) to 40 rpm in 2 min. One trial lasted for a maximum of 500 s. Between each trial, the mice were allowed to rest for 30 min.

#### 2.2.5. Morris Water Maze Task

Morris water maze tasks were performed as previously described [18]. Briefly, the mice were tested in a black circular pool with a diameter of 180 cm and a height of 60 cm that was theoretically divided into four equal quadrants. The 13 cm diameter escape platform, which was submersed 1 cm below the water surface, was put in the center of one quadrant. In each training trial, the mice explored freely in the maze for up to 90 s. Those who did not reach the escape platform within 90 s were gently led to it by the experimenter. After each trial, the mouse was left on the escape platform for another 20 s. The training was conducted 4 times a day for 4 consecutive days. Between each trial, the interval was more than 30 min. Twenty-four hours after the last training, a probe trial was conducted for 90 s without the escape platform. The swimming speed, the first latency to the platform (target) zone, and the distance traveled until the first entry into the target zone were measured to evaluate spatial memory.

### 2.3. Transcriptome Sequencing

The brain transcriptome was sequenced by Vazyme Biotech Co., Ltd. (Nanjing, China) and described previously [18]. In brief, the brains of 21-day old female mice (*n* = 4 per group) were collected from 2 litters (2 mice per litter) with different parents. Total RNA was isolated from the cerebrum using Trizol (Invitrogen, Carlsbad, CA, USA) following the manufacturer’s instructions and cleaned up by RNeasy spin columns (Qiagen, Valencia, CA, USA). The concentration and purity of isolated RNA were determined by a Qubit^®^ RNA Assay Kit (Life Technologies, Carlsbad, CA, USA) and NanoDrop^®^ spectrophotometers (Thermo Fisher Scientific, Waltham, MA, USA), respectively. The integrity of isolated RNA was determined by a LabChip GX system (Caliper, Newton, MA, USA). The quality score of RNA ranges between 7.6 and 8.4. The sequencing libraries were prepared using VAHTS mRNAseq v2 Library Prep Kit for Illumina R (Vazyme, Nanjing, China) following the manufacturer’s recommendations. The sequencing was performed on an Illumina HiseqTM X Ten platform (Illumina, San Diego, CA, USA) with 150 bp paired-end reads. Low quality reads and those containing adapter or ploy-N were removed from the raw reads. Then the clean reads were aligned to the reference mouse genome (mm10) using TopHat (version 2.1.1) and assembled using the reference-based assembler Cufflinks (v2.2.1). The fragments per kilobase of transcript sequence per millions base pairs, mapped (FPKM) for coding genes, was calculated by Cuffdiff (v1.3.0). Comparing FPKM values of the control and 2.5 × FA mice brains, transcripts with a fold change > 2 and an adjusted *p* < 0.05 were considered as significantly differentially expressed. Gene ontology (GO) enrichment analysis of DEGs was conducted with GO::TermFinder. Kyoto Encyclopedia of Genes and Genomes (KEGG) analyses for the DEGs were performed using the Database for Annotation, Visualization, and Integrated Discovery (DAVID) bioinformatic resources (https://david.ncifcrf.gov/, accessed on 25 August 2017).

### 2.4. qRT-PCR

Cerebral RNA (*n* = 3) was extracted from 2 litters with different parents. HiScript II Q RT SuperMix for qPCR (with gDNA wiper) (Vazyme, Nanjing, China) was used to reverse transcribed equal amount of RNA of each sample to complementary DNA. Real-time PCR was conducted with AceQ qPCR SYBR^®^ Green Master Mix (Vazyme, Nanjing, China) on a LightCycler^®^ 480 (Roche Diagnostics, Basel, Switzerland).

The amplifying conditions for cDNA were as follows: denaturation at 95 °C for 10 min, 45 cycles of 95 °C for 10 s and 60 °C for 30 s, followed by 95 °C for 10 s, 65 °C for 60 s, 97 °C for 1 s and 37 °C for 30 s. Quantitation of the relative levels of mRNA were carried out by the comparative 2^−ΔΔCt^ method and normalized to Ribosomal Protein S18 (RPS18) levels (B661301, Sangon Biotech, Shanghai, China). Primer sequences are listed in Table 1.

### 2.5. Western Blot

Mice cerebrums (*n* = 6) from 2 litters (3 females per litter) with different parents were homogenized in a 9-fold volume of ice-cold brain lysis buffer (50 mM Tris-HCl, pH 7.4, 2.0 mM EDTA, 8.5% sucrose and 10 mM β-mercaptoethanol) containing a protease inhibitor cocktail (Roche, Basel, Switzerland). Protein concentration of the samples was measured by a Pierce™ 660 nm protein assay kit (Thermo Fisher Scientific). The same levels of protein in each sample were loaded and separated by sodium dodecyl sulfate-polyacrylamide gel electrophoresis (SDS-PAGE). The protein was then transferred onto polyvinylidene difluoride (PVDF) membrane. The membrane was blocked with 5% skim milk in Tris-buffered saline (TBS, 150 mM NaCl, 50 mM Tris-HCl, pH 7.4) for 30 min and incubated with rabbit anti-Sult1a1 antibody (1:500, bs-6283R, Bioss Antibodies, Beijing, China) overnight at room temperature. Then the membrane was rinsed three times with TBST (TBS containing 0.05% Tween-20) and incubated with peroxidase affinipure goat anti-rabbit IgG (111-035-144) (Jackson ImmunoResearch, West Grove, PA, USA) for 2 hr at room temperature, and then developed with an Enhanced chemiluminescence (ECL) kit (Thermo Fisher Scientific) and exposed to X-ray film (Carestream Health, White City, OR, Canada). The intensity of bands was quantified using Multi Gauge software (Fuji Film, Tokyo, Japan).

### 2.6. Statistical Analysis

Data were presented as mean ± standard error of the mean (SEM). The statistical significance was analyzed using GraphPad Prism 8 (GraphPad Software Inc., San Diego, CA, USA) by a two-way analysis of variance (ANOVA) followed by a post hoc analysis using the Bonferroni’s multiple comparison test or the unpaired two-tailed Student’s *t* test. *p* < 0.05 was considered statistically significant.

## 3. Results

### 3.1. Excess FA Supplementation throughout Pregnancy and Lactation Modifies Behaviors in the Adult Female Offspring

**Figure 1:**
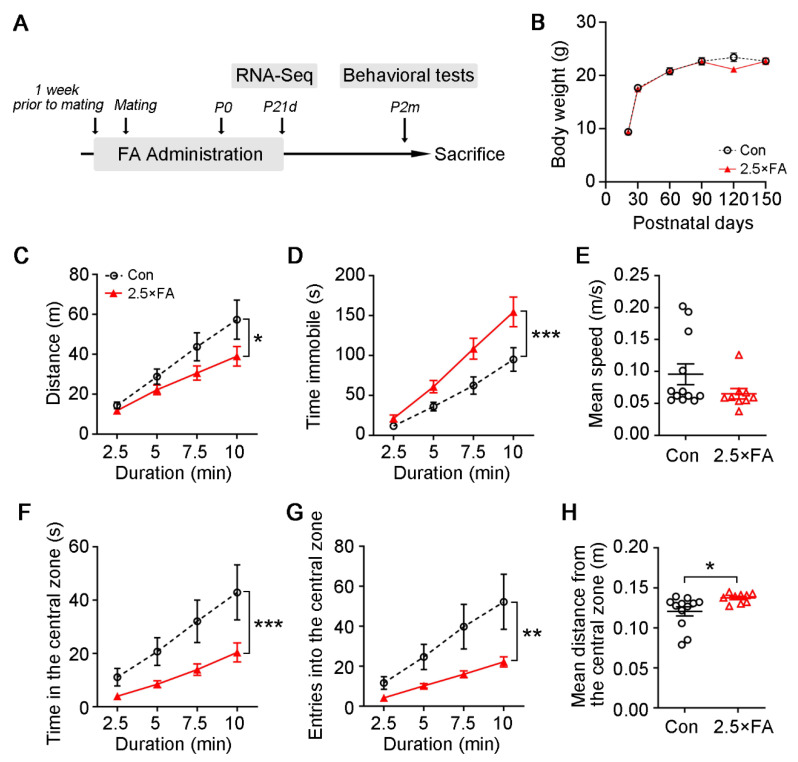
Effect of excess FA intake on body weight and the performance in open field test of the female offspring. (**A**) Schematic of the study design. (**B**) Control (Con) and 2.5 × FA female mice were weighed from postnatal day 21 to 150. (**C**–**H**) In the open field test, the distance traveled by the female offspring in the arena (**C**), the immobile time (**D**), the mean speed (**E**), the time spent in the central square (**F**), the numbers of entries into the central square (**G**) and the mean distance from the boundary of the central zone (**H**) were measured. Con, *n* = 12; 2.5 × FA, *n* = 9. *, *p* < 0.05; **, *p* < 0.01; ***, *p* < 0.001.

The mice were fed with drinking water dissolved with 3.75 mg/L FA (about 2.5 times the dietary requirement) from one week before mating, and throughout the mating, gestation and lactation periods (Figure 1A). The female offspring were weaned to normal drinking water at postnatal day 21 (P21d). The body weight of female offspring (2.5 × FA hereafter) was tracked for 5 months, and there was no difference when compared with the control (Figure 1B, FA→*F*_1, 114_ = 1.418, *p* = 0.2362; Day→*F*_5, 114_ = 138.9, *p* < 0.0001; Day × FA→*F*_5, 114_ = 1.058, *p* = 0.3873), suggesting that early-life FA supplementation did not affect the physical growth of the female offspring.

Two-month old 2.5 × FA female mice were subjected to several behavioral tests. First, their locomotor/exploratory activities and anxiety-like behavior were assessed by the open field test. Although the mean speed of 2.5 × FA mice showed no difference from that of the control (Figure 1E, *p* = 0.1465), the overall distance that 2.5 × FA mice traveled in the open field decreased (Figure 1C, Duration *F*_3, 76_ = 14.50, *p* < 0.0001; FA *F*_1, 76_ = 6.618, *p* = 0.0120; Duration × FA *F*_3, 76_ = 0.7745, *p* = 0.5118), and their immobile time increased significantly (Figure 1D, Duration *F*_3, 76_ = 37.44, *p* < 0.0001; FA *F*_1, 76_ = 21.02, *p* < 0.0001; Duration × FA *F*_3, 76_ = 2.121, *p* = 0.1045). The 2.5 × FA mice spent less time in the central zone of the open field (Figure 1F, Duration *F*_3, 76_ = 5.928, *p* = 0.0011; FA *F*_1, 76_ = 12.23, *p* = 0.0008; Duration × FA *F*_3, 76_ = 0.6138, *p* = 0.6081) and entered the central zone less than the control (Figure 1G, Duration *F*_3, 76_ = 5.179, *p* = 0.0026; FA *F*_1, 76_ = 11.52, *p* = 0.0011; Duration × FA *F*_3, 76_ = 0.8008, *p* = 0.4972). Moreover, 2.5 × FA mice were farther away from the boundary of the central zone than the control mice (Figure 1H, *p* = 0.0263). These results showed that excess FA supplementation impaired exploratory activity and increased anxiety-like behavior of the adult female offspring.

**Figure 2:**
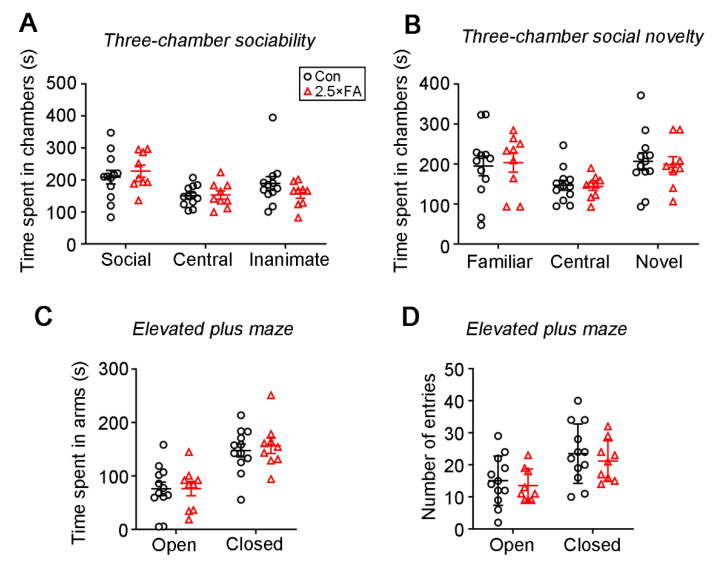
2.5 × FA did not affect the sociability and anxiety-like behavior of the female offspring. (**A**,**B**) In the three-chamber social approach and social novelty test, the time of mice spent in different chambers during the sociability phase (**A**) or the social novelty phase (**B**) were recorded. (**C**,**D**) In the elevated plus maze, the time of mice spent in the closed or open arms (**C**) and the number of entries (**D**) into the closed or open arms were recorded. Con, *n* = 12; 2.5 × FA, *n* = 9.

Previously, we reported that excess FA impaired social preference of the adult male offspring in the three-chamber sociability test; that is, 2.5 × FA males spent less time in the chamber with a social target (a stranger mouse) [18]. Here, we performed the three-chamber social approach and social novelty test on 2.5 × FA female mice and found no obvious abnormality (Figure 2A,B). When compared with the control, 2.5 × FA mice spent a similar time in the chamber containing the social target or an inanimate target during the sociability phase (Figure 2A, FA *F*_1, 19_ = 1.349, *p* = 0.2598; Chamber *F*_2, 38_ = 5.138, *p* = 0.0106; Chamber × FA *F*_2, 38_ = 0.7914, *p* = 0.4606), and in the chamber with a familiar mouse or a novel target (a new unfamiliar mouse) during the social novelty phase (Figure 2B, FA *F*_1, 19_ = 0.0488, *p* = 0.8275; Chamber: *F*_2, 38_ = 3.498, *p* = 0.0403; Chamber × FA *F*_2, 38_ = 0.0569, *p* = 0.9448), indicating that FA did not affect the social performance of the female offspring.

Next, the mice were assessed for anxiety-like behavior using the elevated plus-maze. The residence time and number of entries of 2.5 × FA mice in the open arms or in the closed arms was not different from that of the control mice (Figure 2C, FA *F*_1, 38_ = 0.1213, *p* = 0.7295; Arm *F*_1, 38_ = 33.44, *p* < 0.0001; FA × Arm *F*_1, 38_ = 0.1059, *p* = 0.7466; Figure 2D, FA *F*_1, 38_ = 0.6638, *p* = 0.4203; Arm *F*_1, 38_ = 11.86, *p* = 0.0014; FA × Arm *F*_1, 38_ = 0.0258, *p* = 0.8733), suggesting that 2.5 × FA supplementation did not induce anxiety in the female offspring in the elevated plus-maze.

**Figure 3:**
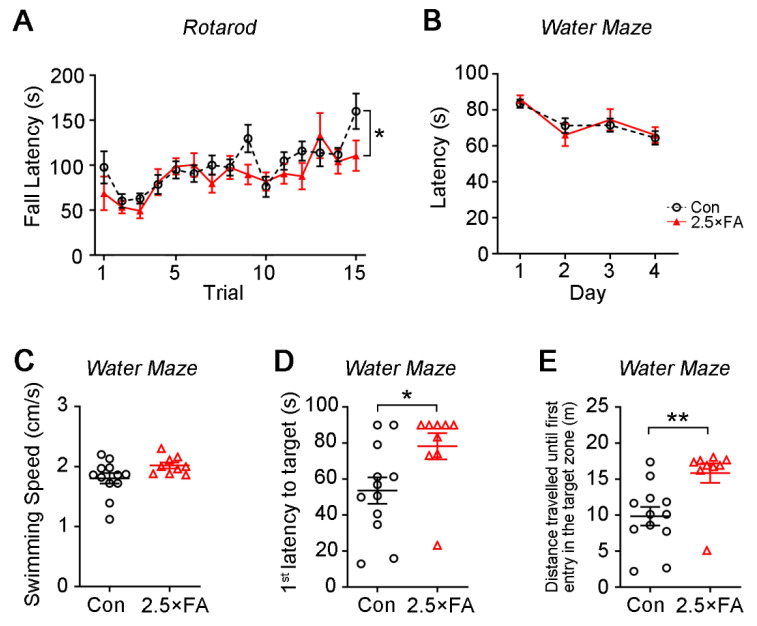
The motor learning and spatial memory abilities were impaired in 2.5 × FA mice. (**A**) Motor learning and coordination of the mice was assessed by rotarod. (**B**–**E**) Spatial learning and memory of 2.5 × FA mice were assessed by Morris water maze test. (**B**) During the training days, the time it took the mice to reach the escape platform (latency) was recorded. Twenty-four hours after the last training day, the probe trials were performed. Swimming speed of the mice (**C**), the first latency (**D**) and the travel distance to reach the escape platform (target) (**E**) were recorded. Con, *n* = 12; 2.5 × FA, *n* = 9. *, *p* < 0.05; **, *p* < 0.01.

Motor learning and coordination of the mice were analyzed by an accelerating rotarod. Although both groups showed an increasing latency to fall from the rotarod with the increase of trials, 2.5 × FA mice displayed less improvement in their performance (Figure 3A, FA *F*_1, 285_ = 5.679, *p* = 0.0178; Trail *F*_14, 285_ = 5.575, *p* < 0.0001; Trial × FA *F*_14, 285_ = 1.121, *p* = 0.3386), implying that excess FA impaired the motor coordination of the female offspring.

We used the Morris water maze to assess the spatial learning and memory of 2.5 × FA female mice. During the training days, 2.5 × FA and control mice spent similar time before finding the escape platform (Figure 3B, FA *F*_1, 76_ = 0.0268, *p* = 0.8703; Day *F*_3, 76_ = 8.384, *p* < 0.0001; Day × FA *F*_3, 76_ = 0.4246, *p* = 0.7359). In the probe trial, no significant difference was found in the swimming speed between 2.5 × FA and control mice (Figure 3C, *p* = 0.0652). However, 2.5 × FA mice took more time (Figure 3D, *p* = 0.0306) and traveled a longer distance (Figure 3E, *p* = 0.0052) to reach the target zone, displaying deficiency of spatial memory.

Taken together, these data revealed that excess FA supplementation during pregnancy and lactation period increased anxiety-like behavior and reduced the exploration, motor coordination and spatial memory of the adult female offspring, without affecting their general growth, athletic ability, sociability and preference for social novelty.

### 3.2. Excess FA Alters Brain Transcriptome of the Female Offspring at Weaning

**Figure 4:**
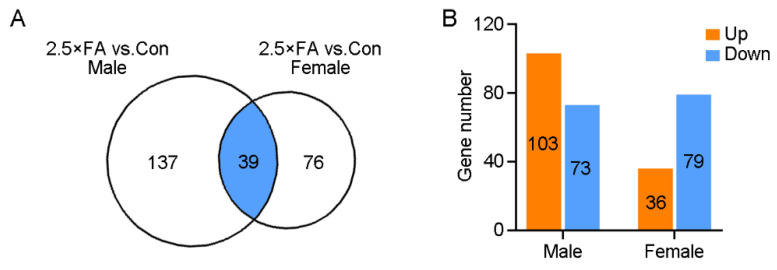
The transcriptome was altered in the brains of 2.5 × FA mice at P21d. RNA extracted from the cerebrum of control and 2.5 × FA mice (*n* = 4) was analyzed by transcriptome sequencing. (**A**) Venn diagram indicates the number of DEGs that are unique in each comparison or shared (blue) between groups. (**B**) The number of up-regulated (orange) or down-regulated (blue) genes in 2.5 × FA male or female mice brain.

We previously identified 176 DEGs in the brains of FA-exposed male mice at P21d using RNA sequencing (103 up-regulated and 73 down-regulated) [18]. In the present study, 2.5 × FA female brains were also collected at P21d and subjected to RNA sequencing. Analysis of the brain transcriptome revealed 115 DEGs in 2.5 × FA mice when compared to the control female brains (36 up-regulated genes and 79 down-regulated genes, Figure 4A,B, Supplementary Table S2). The number of DEGs induced by excess FA exposure in female brain is less than two-thirds of that in male brain, implying that the female brain transcriptome was less sensitive to the same dosage of FA. Thirty nine genes were differentially expressed in both male and female 2.5 × FA brains (16 up-regulated and 23 down-regulated, Figure 4A, Supplementary Table S3). The differential changes of the brain transcriptome might explain distinct behavioral abnormalities of female and male mice upon early-life FA exposure.

### 3.3. Analysis of Pathway and Gene Ontology Enrichment of the DEGs in 2.5 × FA Brains

**Figure 5:**
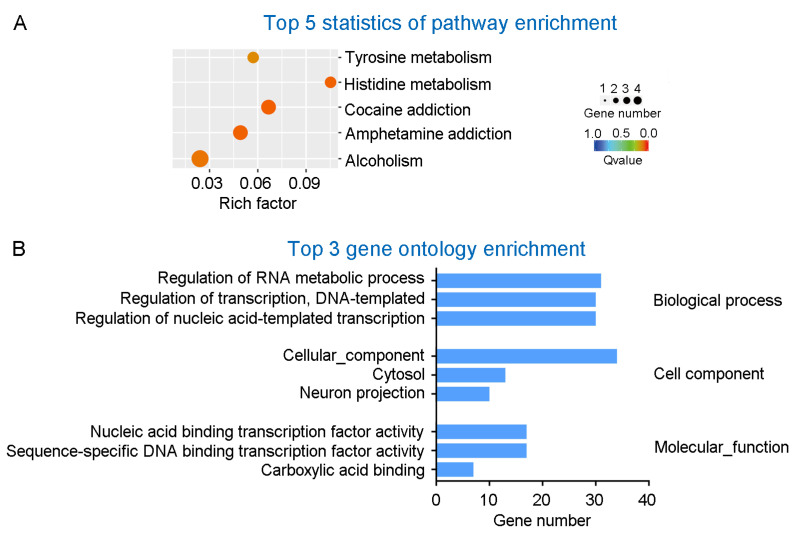
KEGG and gene ontology (GO) functional enrichment analysis of DEGs in 2.5 × FA female brains. (**A**) Top 5 statistics of KEGG pathway enrichment for the DEGs in 2.5 × FA female brains. (**B**) Top 3 statistics of GO term enrichment for the DEGs in 2.5 × FA female brains.

The KEGG (Kyoto Encyclopedia of genes and Genomes) analysis of the 115 DEGs in the brains of female 2.5 × FA mice displayed five major enrichment pathways involving alcoholism (Pathway ID: ko05034), amphetamine addiction (ko05031), cocaine addiction (ko05030), histidine metabolism (ko00340) and tyrosine metabolism (ko00350) (Figure 5A). Among these pathways, alcoholism, amphetamine addiction and cocaine addiction pathways were also enriched in 2.5 × FA male brains (Table 2).

The DEGs were then categorized according to the gene ontology (GO) analysis. For 2.5 × FA females, the top significantly enriched GO terms in the category of biological process, cell component and molecular function were regulation of RNA metabolic process (GO ID: 0051252), cellular component (GO: 0005575) and nucleic acid binding transcription factor activity (GO: 0001071), respectively (Figure 5B). However, the GO enrichment of DEGs in 2.5 × FA female and male brains showed considerable differences (Table 3).

### 3.4. The Expression of Genes Enriched in the GO Category of Cellular Component Were Changed in 2.5 × FA Brains at Weaning

**Figure 6:**
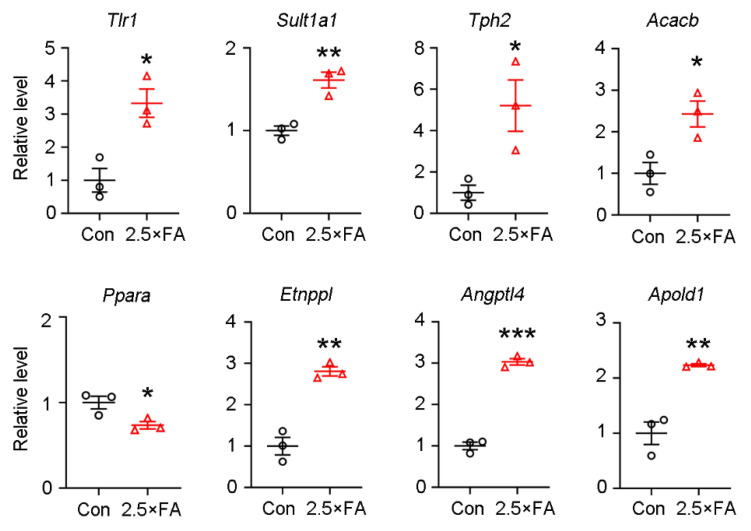
DEGs enriched in the GO category of cellular component were validated by qRT-PCR. The relative mRNA expression level of *Tlr1*, *Sult1a1*, *Tph2*, *Acacb*, *Ppara*, *Etnppl*, *Angptl4* and *Apold1* genes in control and 2.5 × FA brains (*n* = 3) were determined by qRT-PCR. The levels of ribosomal protein S18 (RPS18) mRNA were used for normalization. *, *p* < 0.05; **, *p* < 0.01; ***, *p* < 0.001.

Since most DEGs were enriched in the GO category of cellular component (Figure 5B), we further validated the transcription levels of these genes by qPCR and found that the mRNA levels of *Tlr1* (*toll like receptor 1*) (*p* = 0.0138), *Sult1a1* (*sulfotransferase family 1A, phenol-preferring, member 1*) (*p* = 0.0052), *Tph2* (*tryptophan hydroxylase 2*) (*p* = 0.0311), *Acacb* (*acetyl-CoA carboxylase beta*) (*p* = 0.0246), *Etnppl* (*ethanolamine-phosphate phospho-lyase*) (*p* = 0.0017), *Angptl4* (*angiopoietin like 4*) (*p* < 0.0001) and *Apold1* (*apolipoprotein L domain containing 1*) (*p* = 0.0039) were significantly increased, while the expression of *Ppara* (*peroxisome proliferator activated receptor alpha*) was down-regulated (*p* = 0.0370) in 2.5 × FA brains (Figure 6).

**Figure 7:**
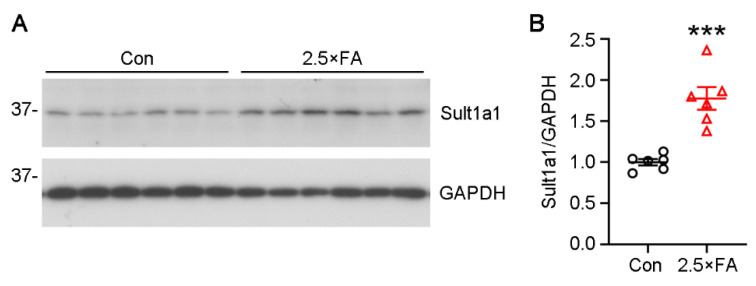
The protein levels of Sult1a1 were elevated in 2.5 × FA brains at P21d. (**A**) The brains of control and 2.5 × FA mice were lysed and analyzed by Western blots, comparing anti-Sult1a1 levels (*n* = 6). GAPDH was used as a loading control. (**B**) The protein levels of Sult1a1 were calculated by normalizing to GAPDH. ***, *p* < 0.001.

The protein expression of 2.5 × FA brains was then analyzed using Western blots. The protein levels of Sult1a1 in 2.5 × FA brains increased by more than 1.5-fold (Figure 7, *p* = 0.0003). These data revealed that excess FA supplement altered the brain transcriptome in female offspring at weaning.

## 4. Discussion

Moderate FA intake from food fortification and vitamin supplementation has been accepted to protect against neural tube defects and other birth defects [2]. However, there is also evidence indicating that excess FA supplementation has side effects on neurodevelopment [19,28,29,30,31]. We have reported that excess FA supplement before and throughout the pregnancy and lactation periods leads to long-term weight gain and abnormal behaviors in adult male offspring, such as deficits in social preference, anxiety-related behavior and abnormal motor and spatial learning abilities [18]. Here, we examined the influence of the same dosage of FA on the behaviors of female offspring and found distinct responses (summarized in Table 4). Unlike the results in males, 2.5 × FA neither affected the general growth nor caused defects in social behavior and performance in the elevated plus-maze of the adult female offspring (Figure 1B and Figure 2). In the accelerating rotarod test, the motor learning of males has been shown to lag behind from the fourth trial [18], while the difference between 2.5 × FA and control females is far less dramatic (Figure 3A). Nevertheless, the anxiety-like behavior and decreased exploration in open field (Figure 1C–H), and the impaired spatial memory in the water maze (Figure 3C–E) were specifically identified in females but not in males. These results showed that excessive FA during pregnancy and lactation had adverse effects on the behaviors of adult female offspring, but the specific effects were different from those in males.

As the fully oxidized form of folate, FA is reduced to tetrahydrofolate by the two-step catalysis of dihydrofolate reductase (DHFR) in vivo, and then to 5-MTHF through methylenetetrahydrofolate reductase (MTHFR). The biologically active 5-MTHF transfers a methyl group to homocysteine (Hcy) to form methionine. The latter is further converted to SAM, the universal methyl group donor. After participating in various methylation reactions, SAM is converted to S-adenosyl-homocysteine (SAH) [32]. Excess FA intake leads to the high levels of unmetabolized FA and total folate in maternal and fetal circulation [33] as well as in breast milk [4]. Furthermore, FA supplementation has been shown to elevate the SAM/SAH ratio in human neuroblastoma SH-SY5Y cells [34] and human plasma [35]. At present, we cannot distinguish whether the abnormal behaviors and brain gene expression of the mice offspring are caused by unmetabolized FA or excessive methyl donors, or both. If the levels of unmetabolized FA, reduced folates, SAM, SAH and the SAM/SAH ratio could be measured in the blood and brains of 2.5 × FA mice, it may help to better understand this issue.

The gender-specific influences of excess FA on behavioral abnormalities have been reported by other groups. Pregnant mice fed with 4 mg/kg diet of FA induced hyperactivity of male but not female offspring [13]. Maternal over-supplementation with FA has been associated with attentional dysfunction in children, particularly in boys [19]. The potential mechanism for the different behavioral responses between genders might be the sexual variation in FA metabolism. For example, the expression of MTHFR, a key enzyme that generates 5-MTHF for Hcy remethylation in the folate and Hcy metabolic pathways, was higher in male liver than in the female liver. 10 mg FA/kg diet (5 times the recommended amount) increased the ratio of phosphorylated MTHFR and decreased glycerophosphocholine and sphingomyelin in the brains of male but not female offspring [29]. Whether the expression and activity of one-carbon metabolism enzymes, such as DHFR, MTHFR, serine hydroxymethyltransferase (SHMT) and methionine synthetase (MS), are different between males and females in the liver and/or the brain at various embryonic and postnatal ages, and how the enzymes respond to excess FA intake, are worthy of further investigation.

Excess maternal FA has been shown to modulate gene expression levels and DNA methylation profiling in the cerebellum, depending on the sex of mouse offspring [13,17,36]. In the present study, the transcriptome of FA-exposed mouse brain also exhibited gender-related differences (Table 2 and Table 3). The male and female differences of brain transcriptome alternations were mainly concentrated in two areas: gene number and interrupted processes. Firstly, when compared with males, the total number of DEGs in the brain of weaning 2.5 × FA female mice decreased by one-third, and the number of up-regulated genes decreased by two-thirds (Figure 4A,B). Secondly, the DEGs in males were mostly enriched in the GO biological process category-“Cell–cell signaling”; while in females, the largest number of genes were concentrated in the category “regulation of RNA metabolic process” (Figure 5B, Table 3), which contains various zinc finger proteins. Unlike in males, there were no significant changes in the expression of *Fos* and associated genes [18] in 2.5 × FA female brains. Instead, several genes in the GO cell component category-“cellular component” were dysregulated in female brains. In general, the differences in the number and categories of DEGs caused by 2.5 × FA in female and male brains may contribute to distinct behavioral responses between genders. Whether gender and/or gene polymorphism-induced differences in FA metabolism [37,38] also lead to variant neurodevelopmental responses upon excess early-life FA exposure in humans is worth further investigation.

Transcriptome analysis of the whole brain in this study provided a general view of gene expression changes caused by excess FA. Nevertheless, it is also necessary to focus on specific brain regions that are relevant to FA-induced behavioral abnormalities. The performance of mice in the open field is mainly related to brain regions such as the amygdala [39], hypothalamus [40] and prefrontal cortex [41,42]. The cerebellum plays a role in sensorimotor functions, balance control as well as spatial memory and other cognitive functions [43]. The hippocampus functions in spatial navigation, the forming, storing and retrieval of episodic memory and the processing of many other types of memory [44]. Further studies on the gene expression in these specific brain regions could provide more mechanisms for the changes in mouse behavior caused by excess FA.

The transcription of *Sult1a1, Tlr1*, *Tph2*, *Ppara* and *Acacb* gene were exclusively increased in 2.5 × FA female brains. We detected the elevated expression of the Sult1a1 protein, but no significant changes in Tlr1 and Tph2 proteins (data not shown), which may be due to the limited sensitivity of Western blots and/or the spatially restricted expression of these proteins. Sult1a1 catalyzes the sulfonation of a wide range of endogenous metabolites, drugs and xenobiotics, such as neurotransmitters, iodothyronines, catechins, estrogens and hydroxylated aromatic amines [45,46]. It is highly expressed in liver, kidney and heart of C57BL/6 mice and the expression in females is significantly higher than that in males [47]. Sult1a1 expression is also detected in wild-type mice brains in both neurons and glial cells [48] without apparent sex differences [47]. Sult1a1 has been reported to regulate neurotransmitter metabolism through sulfonating monoamines (e.g., dopamine) in vivo [49,50,51]. Tph2 is the rate-limiting enzyme of 5-hydroxytryptamine synthesis in the brain. In most psychiatric diseases, the neurotransmitter serotonin shows an increase in the brain and is considered to be a major therapeutic target, implying that activation of TPH2 would be associated with the pathogenesis of human psychiatric disorders [52]. Accordingly, we speculate that excess FA-induced overexpression of Sult1a1 and/or Tph2 might disturb neurotransmitter metabolism, and therefore contribute to neurodevelopmental disorders.

Toll like receptors (TLRs), which localize on the cell surface or in intracellular vesicular compartments, play an essential role in innate immune responses against pathogens. TLR1 forms heterodimers with TLR2 and is activated in response to bacterial infections, during embryonic brain development or in neuropathogenesis [53,54,55]. Postnatal activation of TLR1/2 but not TLR2/6 heterodimer impairs the performance of adult mice in the Morris water maze, suggesting that early activation of TLR1/2 confers long-term consequences on hippocampus-dependent spatial learning and memory [54]. PPARα is a metabolic regulator involved in lipid metabolism. Knockout of *Ppara* leads to schizophrenia-like phenotypes, including repetitive behavior and lowered prepulse inhibition, and impaired synaptogenesis in the cortex of mice [56]. Activation of TLR1/2 by the agonist peptidoglycan suppressed mRNA, protein and activity of PPARα [57]. Whether excess early-life FA exposure induced by the behavioral deficiencies in adult female mice relies on the Tlr1-dependent inhibition of PPARα remains to be further elucidated. Besides, it has been reported that PPARα mRNA levels are significantly decreased in the liver of rat pups fed vitamin B12-deficient but not FA-deficient diets [58]. Here, we showed the expression of *Ppara* was down-regulated in 2.5 × FA brains. Thus, it is worth exploring whether the expression of *Ppara* in the liver of 2.5 × FA female changes, whether the change is consistent with that found in the brain and the potential mechanisms.

Among the DEGs induced by excess FA intake, the mRNA levels of *Etnppl, Angptl4* and *Apold1* were significantly decreased in both male and female 2.5 × FA mice brains (Figure 6, Supplementary Table S3) [18]. *Etnppl*, a gene specifically expressed in astrocytes [59], is involved in the catabolism of phosphoethanolamine, which is implicated in membrane synthesis [60]. *Apold1* is mainly expressed in endothelial cells [61] and functions in lipid binding, transportation and localization [62]. *Angptl4* is usually highly expressed in adipose tissue and is known as a fasting-induced inhibitor of lipoprotein lipase [63]. In the brain, Angptl4 is secreted by astrocytes under hypoxia and induces brain endothelial cell migration and promotes angiogenesis [64]. These studies suggest that 2.5 × FA might affect lipid metabolism and angiogenesis in the brains of both female and male offspring.

## 5. Conclusions

In conclusion, we found that 2.5 × FA supplementation before and during pregnancy and lactation altered the brain transcriptome of weaning female offspring and impaired the exploratory behavior, motor coordination and spatial memory of female offspring in adulthood. Transcriptome sequencing indicated that excess early-life FA exposure may influence neurodevelopment in multiple aspects, such as neurotransmitter metabolism, immune/inflammation, lipid metabolism and angiogenesis, and involve a variety of cell types including neuron, glial cell and endothelial cell. Therefore, how excess FA mediates the differential gene expression in the brain, how FA-triggered alterations of infant gene expression contribute to long-term influences on the behaviors of adult offspring and whether excess FA supplementation throughout pregnancy and lactation has similar effects on human behaviors deserve more intensive investigation.

## Figures and Tables

**Table 1:** Sequences of qRT-PCR primers.

Gene	Forward Sequence	Reverse Sequence	Amplicon Length (bp)
*Tlr1*	GAGGCATGAAGAGAGCGGAA	TAGGGGTGTCCACAATTGCC	292
*Sult1a1*	GCTAGATAAGTGTGGCCGGG	TTCGGGCAACGTAGATCACC	194
*Tph2*	CAATCGAGTTCGGCCTTTGC	CTGCGTGTAGGGGTTGAAGT	275
*Acacb*	CCGCTCAAGATCGAGGAGTC	ATGCAGGCTACCTTGCTTGT	255
*Ppara*	TGTGAACTGACGTTTGTGGC	CCACAGAGCGCTAAGCTGT	70
*Etnppl*	CGGTCATGTGCGAGCTCTAT	GTCGTGGAGGAAGCGAGAAT	263
*Angptl4*	GGGGACCTTAACTGTGCCAA	CTGCAGAGGATAGTAGCGGC	165
*Apold1*	CCGTCCTGAAGGCCAAGATT	AGAAAAACAACGCTGCGTCC	168

**Table 2:** Top 5 statistics of KEGG pathway enrichment for DEGs in the brains of 2.5 × FA female and male mice.

	Female	Male
1	Alcoholism	Amphetamine addiction
2	Amphetamine addiction	Dopaminergic synapse
3	Cocaine addiction	Cocaine addiction
4	Histidine metabolism	Alcoholism
5	Tyrosine metabolism	Neuroactive ligand–receptor interaction

**Table 3:** Top 5 statistics of GO enrichment for DEGs in the brains of 2.5 × FA female and male mice.

Biological Process	Female	Male
1	Regulation of RNA metabolic process	Cell-cell signaling
2	Regulation of transcription, DNA-templated	Muscle tissue development
3	Regulation of nucleic acid-templated transcription	Feeding behavior
4	Phenol-containing compound metabolic process	Positive regulation of amine transport
5	Phenol-containing compound biosynthetic process	Positive regulation of anion transport
**Cellular component**	**Female**	**Male**
1	Cellular_component	Plasma membrane part
2	Cytosol	Cell projection
3	Neuron projection	Neuron projection
4	Cytoplasmic membrane-bounded vesicle	Neuron part
5	Synapse part	Integral component of plasma membrane
**Molecular function**	**Female**	**Male**
1	Sequence-specific DNA binding transcription factor activity	Receptor binding
2	Nucleic acid binding transcription factor activity	Enzyme inhibitor activity
3	Organic acid binding	Hormone activity
4	Carboxylic acid binding	Drug binding
5	Amino acid binding	Neuropeptide hormone activity

**Table 4:** Summary of body weight changes and behavioral results of 2.5 × FA female and male mice.

	2.5 × FA vs. Control	
	Female	Male [18]
Body weight	NS ^a^	Increased
Open field	Decreased exploration Increased anxiety	NS ^a^
Three-chamber sociability	NS ^a^	Decreased sociability
Three-chamber social novelty	NS ^a^	NS ^a^
Elevated plus-maze	NS ^a^	Increased anxiety
Accelerating rotarod	Impaired motor learning	Impaired motor learning
Morris water maze	Impaired spatial memory	Delay in spatial learning

^a^ No significant difference was identified.

## Data Availability

The data presented in this study are available on request from thecorresponding author. The data are not publicly available due to confidentiality reasons.

## Supplementary Materials

The following are available online at https://www.mdpi.com/article/10.3390/nu14010066/s1, Table S1: Diet formula, Table S2: Differential expressed genes in 2.5 × FA compared to control female mice brains at P21d, Table S3: Genes differentially expressed in both 2.5 × FA female and male brains.
